# Social service providers’ perspectives on caring for structurally vulnerable hospital patients who use drugs: a qualitative study

**DOI:** 10.1186/s12913-022-08498-x

**Published:** 2022-09-08

**Authors:** Nicole D. Gehring, Kelsey A. Speed, Kathryn Dong, Bernie Pauly, Ginetta Salvalaggio, Elaine Hyshka

**Affiliations:** 1 School of Public Health, University of Alberta, Edmonton, AB Canada; 2 Inner City Health and Wellness Program, Royal Alexandra Hospital, Edmonton, AB Canada; 3 Faculty of Medicine and Dentistry, University of Alberta, Edmonton, AB Canada; 4 School of Nursing, University of Victoria, Victoria, BC Canada

**Keywords:** Social needs, Social work, Social services, Structural vulnerability, Illegal drugs, Houseless, Acute care, Qualitative research

## Abstract

**Background:**

People who use drugs and are structurally vulnerable (e.g., experiencing unstable and/or lack of housing) frequently access acute care. However, acute care systems and providers may not be able to effectively address social needs during hospitalization. Our objectives were to: 1) explore social service providers’ perspectives on addressing social needs for this patient population; and 2) identify what possible strategies social service providers suggest for improving patient care.

**Methods:**

We completed 18 semi-structured interviews with social service providers (e.g., social workers, transition coordinators, peer support workers) at a large, urban acute care hospital in Western Canada between August 8, 2018 and January 24, 2019. Interviews explored staff experiences providing social services to structurally vulnerable patients who use drugs, as well as continuity between hospital and community social services. We conducted latent content analysis and organized our findings in relation to the socioecological model.

**Results:**

Tensions emerged on how participants viewed patient-level barriers to addressing social needs. Some providers blamed poor outcomes on perceived patient deficits, while others emphasized structural factors that impede patients’ ability to secure social services. Within the hospital, some participants felt that acute care was not an appropriate location to address social needs, but most felt that hospitalization affords a unique opportunity to build relationships with structurally vulnerable patients. Participants described how a lack of housing and financial supports for people who use drugs in the community limited successful social service provision in acute care. They identified potential policy solutions, such as establishing housing supports that concurrently address medical, income, and substance use needs.

**Conclusions:**

Broad policy changes are required to improve care for structurally vulnerable patients who use drugs, including: 1) ending acute care’s ambivalence towards social services; 2) addressing multi-level gaps in housing and financial support; 3) implementing hospital-based Housing First teams; and, 4) offering sub-acute care with integrated substance use management.

**Supplementary Information:**

The online version contains supplementary material available at 10.1186/s12913-022-08498-x.

## Background

Structural vulnerability is the manifestation of intersecting political, socioeconomic, and cultural hierarchies that impact the health of individuals and populations [1, 2]. People who use drugs are often structurally vulnerable due to severe socio-political disparities that amplify stigmatization, discrimination, and cultural oppression. Structurally vulnerable patients who use drugs, particularly those experiencing unstable and/or lack of housing, disproportionately access acute care compared to the general public [3, 4]. Hospitalized patients who use drugs are more likely than other hospitalized patients to experience unstable/lack of housing and report acute care as their primary point of healthcare access [5, 6]. Structurally vulnerable patients rely on acute care more often for several reasons, including access barriers (e.g., no identification, health insurance), lack of primary care continuity, and/or prior experiences of stigma and discrimination in healthcare settings [7, 8] that can result in delayed care seeking until health conditions require urgent medical attention. These factors often reinforce acute care as the most accessible and convenient healthcare option.

Conventionally, hospitals provide short-term diagnostic assessment and acute medical treatment. Although structurally vulnerable patients often present to acute care with unmet social needs (e.g., inadequate housing, food insecurity, unemployment, safety concerns, difficulty affording basic needs) [9, 10], acute care systems and providers may not be able to effectively address these determinants of health during hospitalization [6, 11]. Instead, structurally vulnerable patients are frequently discharged back to emergency shelters or onto the street, further compounding health inequities [12]. This is concerning because addressing social needs can improve post-discharge outcomes, decrease readmissions, and shorten the length of hospital stays amongst structurally vulnerable patients who use drugs [13, 14]. For example, provision of housing after hospital discharge is associated with improved health outcomes and sustained housing [15].

The integration of social services within acute care settings is one potential strategy to address the broader social needs of patients. While social service providers in acute care hospitals have specialized training to help meet basic and complex needs of patients, they receive little guidance on how to care for patients who use drugs [16] or those with unstable and/or lack of housing [17], let alone patients experiencing both substance use *and* unstable/lack of housing. There is also limited literature regarding effective social service provision specific to structurally vulnerable patients who use drugs. The majority of research examines addressing social needs for general acute care patients, *or* for those experiencing unstable and/or lack of housing *or* those who use drugs, exclusively. This is problematic given the high prevalence of substance use disorders and unstable and/or lack of housing amongst structurally vulnerable populations [18, 19] and the unique challenges associated with supporting this patient population effectively.

Patients who use drugs and experience unstable and/or lack of housing report feeling judged and unwelcomed within hospital settings, and describe futility in the care they are provided [20, 21]. Hospitals also often enforce formal or informal bans on illegal drug use [22, 23]. As a result, patients can hesitate to disclose their drug use or housing status [24, 25]. Nondisclosure leaves these important aspects of health neglected, while disclosure can lead to stigmatized clinical encounters [24, 26]. Effective care for this patient population requires tailored and coordinated interventions that address both housing and drug use simultaneously. However, little research has explored how to respond to barriers impeding the delivery of social services in hospitals, and extant studies focus on the perspectives of social workers only. The views of other professionals who address social needs (e.g., peer support workers, transition coordinators) have received little attention, resulting in a narrow perspective on social service delivery within acute care. We explored the perspectives of social service providers at a large urban acute care hospital on: 1) the barriers and facilitators they face in addressing the social needs of structurally vulnerable patients who use drugs; and 2) if they identified any possible strategies for improving care for this patient population. Our overall aim was to generate knowledge on social service provision that could lead to better integration of social services within acute care to improve health outcomes for this patient population.

## Methods

### Study design

We adopted a focused ethnographic design. Compared to traditional ethnography, focused ethnography is more targeted and time-limited [27, 28]. Focused ethnographies are characterized by: focusing on a distinct issue, problem, or experience within a discrete community or organization; being problem-focused and context-specific; involving a limited number of participants who hold specific and specialized knowledge; developing practical recommendations or solutions; and spanning a limited or episodic period of time [27–30]. Focused ethnography commonly employs semi-structured interviews and often limits or omits participant observation in order to generate rapid data [27–29]. This method is frequently used to study highly fragmented or specialized areas, and has been widely used in a variety of healthcare settings [30]. Given our focus on a specialized healthcare setting with a distinct issue (i.e., social service provision), population (i.e., structurally vulnerable patients who use drugs), and community (i.e., social service providers at an urban acute care hospital) this method was well-aligned with our objectives, and helped to quickly generate practical information directly relevant for improving this patient populations’ social needs. Further, this method allowed us to protect the privacy of a structurally vulnerable patient population by not necessitating direct observations of clinical care on hospital units. We report this study using the consolidated criteria for reporting qualitative research (COREQ; see Additional file 1) [31].

### Study setting

The study was conducted at a large, urban acute care hospital located in Edmonton, Canada. While the hospital serves patients from all over Northern and Western Canada, many reside within the local health services catchment of Edmonton-Eastwood. This catchment area is associated with poorer socioeconomic status compared to the provincial average [32], high drug poisoning deaths [33], and the hospital has a high number of emergency department visits and hospitalizations related to substance use [33].

The hospital offers access to an addiction medicine consult team (AMCT). At the time of the study, the AMCT included addiction medicine physicians, a nurse practitioner, social workers, an addiction counsellor, and peer support workers. The team provides in-hospital consultation services for patients experiencing substance use and unstable/lack of housing, including specialized pain and withdrawal management, substance use treatment, harm reduction, access to personal identification, and income and housing support [34, 35]. Social service providers outside of the AMCT (i.e., unit social workers, transition coordinators) work throughout different areas of the hospital to address social needs, where indicated, to the general patient population. While unit social workers provide a range of social services (e.g., psychosocial assessment, advanced care planning, case management and coordination, discharge planning), transition coordinators are focused on facilitating patient discharge and provide resources and services that promote post-discharge planning. Social service providers are also employed by the provincial Department of Community and Social Services who liaise with hospital staff and patients to provide access to client records from across different ministry income support programs.

This study received ethics approval from University of Alberta’s Health Research Ethics Board as part of a larger evaluation of the AMCT.

### Data collection and participants

The AMCT helped identify potential participants through personal invitations, flyer distribution, and presentations at hospital staff meetings. Interview participants also referred colleagues who might be interested in participating. Of 28 potential participants who were referred to, or contacted by, the study team, 10 were lost to follow-up and 18 provided informed consent and participated in a semi-structured interview. The semi-structured interviews were completed between August 8, 2018 and January 24, 2019. AP was the lead interviewer and had no previous relationships with any of the participants. EH joined AP in three earlier interviews. Given the close collaboration between our research group and the hospital, EH was previously acquainted with two participants. However, EH did not hold any influence over these participants or their employment status, and they were advised that their interview would be confidential. In cases where participants' unique roles might incidentally reveal their identity to readers with knowledge of the hospital, participants were given the option of reviewing and approving their transcript prior to inclusion in the analysis. The interview guide (see Additional file 2), which was pilot tested, explored staff experiences providing social services to patients experiencing substance use and unstable and/or lack of housing. It also explored staff views on bridging patients between hospital and community supports. Interviews were held in a private area of the hospital, audio-recorded, lasted approximately one hour, and were de-identified and transcribed verbatim using pseudonyms for participants.

Participants were social workers (SW; *n* = 8) and other social service providers (SSP; *n* = 10), including peer support workers and transition coordinators. The ‘other’ category was used to protect participant anonymity for social service providers occupying otherwise identifiable positions. Participants were affiliated with the AMCT, the inner-city acute care hospital, and the Ministry of Community and Social Services. Participant recruitment and data collection continued until the research team agreed that the transcripts provided rich data, no new ideas or concepts were emerging from interviews, and preliminary analysis showed thematic saturation [36].

### Data analysis

We used NVivo 12 to manage the data. Consistent with focused ethnography and given the descriptive nature of our qualitative study, we performed content analysis [28]. Content analysis uses a descriptive approach to coding and interpretation [37]. Specifically, we conducted latent content analysis. As opposed to manifest content analysis which typically codes and tallies specific words or ideas, latent content analysis emphasizes coding the underlying meaning of text passages and reviewing data within the context of the entire dataset to categorize patterns in the transcripts [27, 28]. This analytical approach was particularly important given the context-specific nature of our study. Examples of how latent content analysis was applied are described below.

The main analyst (NG) reviewed all transcripts and field notes to generate in-depth familiarity with the data and cultivate a general understanding of emergent ideas, words, phrases, and concepts. The data were then coded inductively (i.e., allowing codes emerge from the dataset [28]) using latent content analysis (i.e., coding the meaning and underlying context of text passages [27, 28]). For example, rather than simply coding for instances of discharging patients back onto the street (e.g., ‘discharging to homelessness’), we coded the context in which participants’ described discharging patients back onto the street (e.g., ‘no medical needs to stay in hospital’, ‘pressure to discharge’, ‘patient not receptive’, ‘patient chooses homelessness’). Field notes for each participant were reviewed again during coding to provide additional context. Considerations and deliberations on emerging codes were detailed in a central document. The preliminary codes and codebook were iteratively refined based on several rounds of feedback from KS and EH. Once the codebook had been established, KS reviewed the coding of a subset of the transcripts for coherence and accuracy, paying particular attention to how the codes considered the context of the text passages, and coding was further refined by NG. The final codebook included contextualized accounts of barriers and facilitators to providing social services to this patient population, participants’ perceptions of potential strategies to improve social service provision, and the influence of the social determinants of health and structural vulnerability in social service provision.

Finally, codes were grouped in relation to the socioecological model outlined by McLeroy et al. (1988) to generate themes. The socioecological model considers the complex interplay between individual (e.g., knowledge, attitudes, skills), interpersonal (e.g., families, friends, social networks), organizational (e.g., social institutions, formal and informal rules and regulations), community (e.g., relationships between organizations), and public policy (e.g., local, state, and national laws and policies) features which influence health behaviours [38]. It is particularly helpful for understanding multiple and interacting determinants of health and developing recommendations for multi-level interventions. Once codes were grouped according to the socioecological model, we examined negative cases (i.e., perspectives that contrasted with more commonly occurring perspectives). Negative cases were reviewed to understand the source of their discrepancy, detailed within the audit trail, and groups were revisited and refined [39]. KS and EH reviewed the groupings to ensure each code fit within assigned categories. Each theme was defined and named to provide a descriptive overview, after which participant quotes were selected to complement each theme description. This consisted of revisiting codes and excerpts in each category in their entirety and choosing participant quotes that were representative of the theme description and broader nuance of each theme. As such, each theme heading includes a participant quote and descriptive overview (i.e., “participant quote”: theme description) for transparency on how the two relate to one another and showcase that the single quote captures the context of the theme description. For example, a participant quote highlighting a holistic approach to social service provision represented the sentiment of participants in that theme who proposed comprehensive socio-structural policy. In addition, participant quotes chosen for theme names were not pre-determined and did not guide any part of the analysis.

Themes were ultimately organized in relation to four of the five context-specific levels of the socioecological model: 1) individual; 2) organization 3) community; and 4) policy levels of influence, based on consideration of the entire dataset. For example, codes that contextualized discharging patients back onto the street, were not necessarily categorized together; ‘patient not receptive’ and ‘patient chooses homelessness’ were categorized at the individual level, whereas, ‘no medical needs to stay in hospital’ and ‘pressure to discharge’ were categorized at the organizational level. While the individual level of the socioecological model typically refers to the individual receiving services personally, this level of influence was adapted to describe how social service providers view individual-level patient barriers. While some interpersonal dynamics between social service providers emerged from our analysis they were not prominent in the main findings of our inductive analysis, and thus no related themes are presented here.

Throughout the analytic process, maintaining and reviewing an audit trail of analytic thoughts, decisions, and reflexivity (i.e., iterative positionality statement in relation to the research topic) helped the main analyst identify and engage with potential investigator bias. In addition, we engaged in ongoing discussions with the research team members and consulted members of a community advisory group of people with lived/living experience of substance use, structural vulnerability, and hospitalization, who confirmed our main findings were in line with their own interactions with social service providers.

## Results

**Fig. 1:**
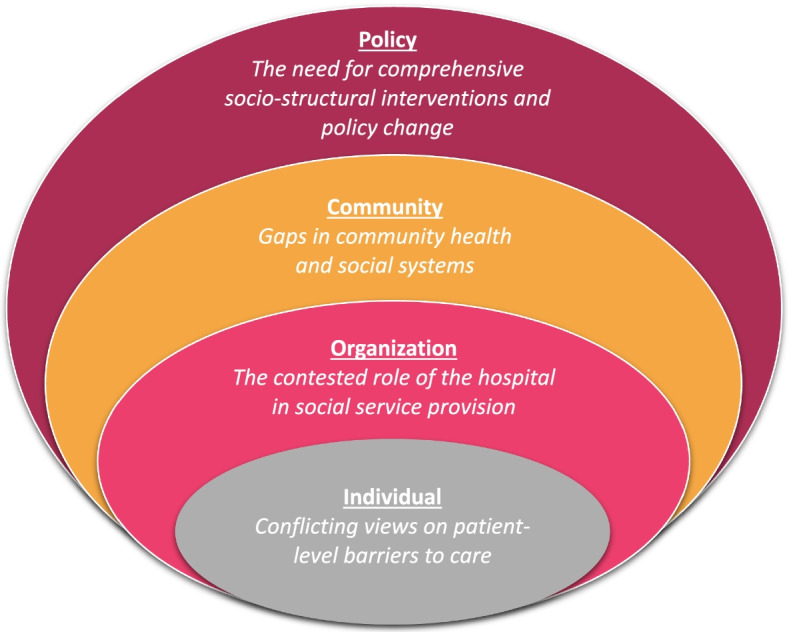
Main themes organized within the socioecological model (figure adapted from [40])

As shown in Fig. 1, four main themes emerged from our qualitative analysis, corresponding to levels of the socioecological model. The main themes are described below from micro- to macro-level of influence: 1) individual; 2) organization; 3) community; and 4) policy.

### “There are people [who] unconsciously or consciously subscribe to an individualist orientation”: conflicting views on patient-level barriers to care

How social service providers conceptualize patient-level barriers to care determines, in part, their approach to addressing needs in practice. Participants in our sample had divergent views, with most emphasizing perceived deficits in patient motivation as the main factor determining unsuccessful social service provision, and a minority highlighting the centrality of structural factors that impede individual patients’ ability to secure income, housing, and other social needs.

Participants attributing patients’ unmet social needs as due to individual factors suggested that some patients “choose” to be houseless, or lack motivation to address their financial circumstances or substance use, and as a result often fail to “follow through” on offers of support. This view was particularly common amongst transition coordinators in our sample. These participants described structurally vulnerable patients who use drugs as “blocking beds” for others with more “legitimate medical needs,” or as “noncompliant” with care plans or hospital rules. These views were often cited as rationale for discharging patients back onto the street. Participants voicing this perspective downplayed the importance of building rapport and trust with structurally vulnerable patients, often expecting patients to access supports on their own, e.g., “put a bunch of papers down…here you go let me know if you need any help” [SSP15]. Another participant explained:

At the end of the day, patients make their own decisions and make their own choices. And if they choose not to help themselves, no matter how much stuff you give them it’s not going to be enough, because they’re still not going to do it. [SSP4]

In contrast, other participants described how patients’ ability to follow through with supports was limited by factors outside of patients’ control. Participants voicing this perspective were largely affiliated with the AMCT. Some participants expressed how post-discharge or outpatient follow-up was challenging because other urgent needs such as “where am I getting my next meal, where am I sleeping tonight” [SSP5] often take priority over keeping scheduled social service appointments. These participants noted that following-up with supports that address social needs could be further hindered by a lack of a phone or transportation and the need to continually focus on securing drugs and avoiding withdrawal. Beyond material challenges, participants outlined how patients find the hospital “inhospitable” and are often discharged when away from the unit for too long, even when they had logical reasons for leaving (e.g., looking for housing, collecting belongings, income generating activities, consuming substances, interacting with peers). Many participants therefore detailed having to allocate a lot of time to building rapport with patients and advocating for patients to stay in hospital in order to adequately address their social needs. For example, a social worker said:

[T]hey may be off the unit because they’re looking for a place…They may have a [substance use] issue that is bringing them off the unit…I’ve had a lot of people be really worried about their stuff and where they’ve stashed their stuff. And they’ve got to go and move it…going and connecting with their peer group out in the smoke pit or things like that…because they’re plus, plus, off unit they kind of get pushed out…So, we have to try and advocate for them to stay in hospital so we can actually help them. [SW6]

The combination of follow-up challenges and the “inhospitable” hospital environment were described as the main reason individual patients “fall through the cracks” [SW7], and ultimately do not get their social needs met.

### “If we view health from a medical model, we’re not understanding the social determinants of health”: the contested role of the hospital in social service provision

At the organization level, participants described tensions in addressing social needs for structurally vulnerable patients given the traditional biomedical approach to acute care. In particular, they discussed the need to frequently turn over available beds and feeling constant pressure to discharge patients back onto the street if patients no longer have acute medical needs. As one social worker shared:

Traditionally hospitals are based on a very medical model…The old school saying that you still hear sometimes on the units is that we’re not here to solve social issues, we’re here to solve medical issues…Being [houseless] is not a medical issue, having no income is not a medical issue so it should not warrant or require that they need to stay in hospital to address this. So, hence, why patients once they’re medically stable, are discharged. I think that social issues are addressed if they impact the hospital stay or the hospital discharge. [SW1]

As a result, most participants outlined how they struggled to provide more than “band-aid” approaches to address patients’ social needs, and being able to only “do something really quick, because they’re being discharged in two days” [SW10].

A few participants were comfortable with the limited range of social services provided in hospital and felt that hospitals should not be responsible for addressing social needs. However, all participants accepting the biomedical model still acknowledged that without providing adequate social services within the hospital, patients will continue to have adverse health and social outcomes. A social worker told us:

I don’t necessarily think that everything needs to be dealt with in an acute care setting. But I think there needs to be some understanding of here’s all these other things that are actually impacting their health and if we don’t address them in some way…overall their health and their wellbeing as a person is not going to get better. [SW8]

In contrast, many participants stressed that hospitals should be responsible for social services because if “we just look at the medical part we are going to wait for them to come back in another week or two” [SW2]. These participants noted that inequities in health and social service access in the community can be alleviated through the hospital because admissions provide an opportunity to reach structurally vulnerable patients who otherwise have limited access to care.

Similarly, participants outlined how the hospital provides a relatively stable environment, which creates an opportunity to comprehensively address social needs. As detailed by a social worker:

It’s actually more productive when they’re in hospital because they have a safe and stable place that they are staying right now that I can find them when I go up to the unit and be able to make progress while they’re in hospital. [SW6]

Others noted that the hospital provides a window to build relationships with patients who otherwise face barriers connecting to care, especially because acute care is often where structurally vulnerable patients access healthcare. For example, one participant told us:

It’s a great time to say here’s an opportunity…especially for [substance use]…so sometimes that window of opportunity is really small, and when they hit that window of opportunity in a hospital, if there’s an opportunity for housing and all those wrap-around services to kind of capitalize on that opportunity. Some people might say it’s a captive audience. [SSP14]

Overall, while some participants felt that acute care was not an appropriate setting to address social needs, most felt that the hospital provides a unique opportunity to provide both medical and social needs to improve outcomes for structurally vulnerable patients who use drugs.

### “It’s almost like they’re set up for failure”: gaps in community health and social systems

Participants noted several gaps in community health and social systems that further challenged their ability to care for this patient population. Most participants discussed a lack of affordable and available housing supports compared to the number of patients in need, resulting in waitlists lasting “close to a year” [SSP4]. Participants noted several other challenges in connecting patients with housing supports, including finding suitable housing, accommodating patient preferences, and patients’ histories with housing supports. Participants outlined how the unique needs of structurally vulnerable patients with current substance use were particularly poorly addressed within mainstream housing programs. For example, one participant said:

Substance use is a huge issue. Even in some of the lodges, for some of our patients who are [houseless], there's only a handful that will take them. Which they’re fantastic but any other lodge that finds out that there’s substance use, is not likely going to take them…[It’s] great to have that [option allowing substance use] but then again, we have a waitlist. [SW8]

Participants further expressed that housing options were restricted for particular groups of structurally vulnerable patients who use drugs, such as women: “Trying to find a…domestic violence women’s shelter who will take somebody with [substance use] issues. I don’t know that that exists” [SW9]. Others described that current shelter and rental housing options for structurally vulnerable patients are typically “rough”, often leaving patients with no viable options. As one participant said:

There are times that because of the existing resources for [houseless] individuals, and how they’re not set up properly, they’re not considered safe, they don't have regulations, if you are somebody who is very vulnerable; it’s not an ideal place. You have people that will refuse to go to them and would rather sleep in a lean-to in the river valley. Like what does that tell you about the way that we treat [this population]? [SSP15]

Finally, participants noted that restrictive and frequently changing criteria for housing supports are a barrier to successfully housing patients. One participant described this challenge by saying:

[Housing] agency’s criteria always change. So, we have to call the same agencies over and over and over again because we never know. So sometimes you get lucky. And somewhere else will have room or make an exception, but there’s nothing easy. [SW9]

Several gaps in financial supports were also identified. Participants noted that income support benefits were insufficient to cover cost of living, requiring patients to have to “choose between…food…or…shelter.” [SSP15]. Participants further added that “if you have a substance [use] problem on top of that, then how do you pay for that?” [SW8]. Participants also described numerous barriers to obtaining and maintaining income support benefits. For example, participants mentioned a cyclical relationship between needing a current address to apply for income support, but also requiring income support to obtain housing. The contradictory nature of obtaining income support was highlighted by two participants who described:

[They] have to have an address so that we can establish residency [to obtain income support]…that’s the piece for individuals that maybe are experiencing homelessness; they do not have an address. [SSP11]

You have to start with their finances. If I don't want to discharge to the street, finances need to be done because in order to get housing you need income. [SW10]

Other barriers to obtaining and maintaining income support benefits included restrictive and convoluted criteria and payment schedules, and unrealistic reporting requirements. For example, one participant said:

[I]t is a lot for people to remember, I mean, my goodness, there are three of us sitting around the table who are educated and articulate and we have a hard time understanding it. So, people with complex needs that are going through [substance use], mental health, trauma, homelessness, whatever it might be, that’s a lot to remember. Even if you’re incredibly…knowledgeable in a lot of different things, when you’re going through a time of crisis, it’s hard to remember those things. [SSP14]

Perhaps most concerning, some participants said that patients residing in shelters are often ineligible for income support, because the provincial government considers their basic needs (e.g., shelter, food) to be met. One participant explained:

The Government…is only responsible for food, shelter, clothing…So, if they’re receiving food and shelter at one of our shelters that the province funds already, to provide a [person] money additionally it could be perceived by some as double dipping. [SSP14]

Gaps in community health and social systems, particularly in housing and income support, were seen as creating intense barriers in providing comprehensive and applicable care for structurally vulnerable patients who use drugs, ultimately exacerbating health and social inequities.

### “We need to look at this from a very holistic perspective”: the need for comprehensive socio-structural interventions and policy change

Several potential policy changes were suggested by participants to help improve acute care experiences, as well as health and social outcomes for structurally vulnerable patients. Many participants said “we would like to have a Housing First team based out of the [hospital]” [SW6] that “would provide a central access point that would prioritize patients leaving acute care” [SW6]. Housing First programs are non-abstinence-based housing initiatives which provide housing to people as quickly as possible, with no preconditions [41]. Participants described several potential benefits to having an in-hospital Housing First team, including: 1) promoting consistency and continuity of care (e.g., mitigate duplication of service offerings, create an easy point of access for inpatients, increase follow-up capacity, enable progress on housing to be made over multiple hospital admissions and/or ambulatory visits); and 2) facilitating the creation of new specialized housing options for patients who use drugs and have co-occurring health conditions. For example, a social worker told us that a Housing First team could start working with acute care patients immediately and allow for better follow up, especially for structurally vulnerable patients with complex health needs:

A Housing First team…that would be aimed towards a specific population that is more vulnerable, with complex health needs…And then leave a small caseload for people that could be easily housed as well so that we’re not missing the whole spectrum right?...there would be an actual team that could go up to the units, grab them and bring them out to look for housing and actually work on that immediately…have that relationship and continue to follow that patient while they’re in housing to help them maintain their housing. [SW1]

Many also described a need for appropriate sub-acute care spaces where patients with medical, social, and substance use needs could wait during hospital-community transitions, because many existing sub-acute facilities often “refuse…inner-city [houseless] patients because of behaviours, because of their substance use, because of mental health” [SW8]. Opening a transitional hospital unit or a community-based sub-acute care facility with a mandate, tailored services, and staff with expertise in the management of patients who use drugs, was seen as one way to prevent discharging medically complex patients back onto the street or keeping them in-hospital while they wait for a space. One social service provider said:

[I]f someone is really ill, it’s hard to find them housing if they’re using [substances]…Even though there is housing for people that use [drugs]. They’re not for people that are also really sick…these are the ones that are stuck in the cracks. [SSP12]

Finally, participants described the need to better identify social determinants of health and substance use within acute care. Not only was this described as a way to enhance existing statistical data on the need for in-hospital Housing First teams and subacute care facilities, but also as a way to identify broader social needs required within acute care and the community. This was particularly important as multi-level interventions addressing broader social needs within existing or proposed housing supports were seen as necessary to better support structurally vulnerable patients who use drugs. Participants told us that multi-level interventions would address personal care skills and support systems since structurally vulnerable patients who use drugs have often lived in extreme poverty for long durations which may limit their ability to maintain housing or income support. For example, a social worker said:

I am talking about people who…have been so entrenched for so many years that they don’t understand how to make a budget, they don’t understand how to grocery shop, they don’t understand how to meal prep…if you take somebody who’s…[used drugs] pretty much most of their life…they have some barriers…come from an unhealthy family system, they don’t have supports and then we finally do get them housed…how are they going to function…They’re not going to know how to maintain this lifestyle now because they’ve never been exposed to it. [SW16]

Taken together, more comprehensive policies and interventions were seen as necessary to address medical, income, and substance use needs concurrently.

## Discussion

To our knowledge, this study is the first to explicitly examine social service providers’ perspectives on addressing the needs of patients who use drugs *and* are experiencing unstable and/or lack of housing within an acute care setting. Specifically, we described the barriers and facilitators to addressing the social needs of structurally vulnerable patients who use drugs and are experiencing unstable and/or lack of housing at the individual, organization, community, and policy levels of influence. Our findings highlight tensions regarding the appropriate scope of social services for structurally vulnerable patients who use drugs, but also the potential for hospitals to play a larger role in providing and advocating for social service provision for this patient population.

Participants had divergent views on patient-level barriers that affected social service provision. Similar findings were reported by Fleming et al. (2017) who found that acute care providers grappled with the complex interplay between structural and individual-level factors, sometimes explaining behaviours as a response to structural conditions, and other times as the result of individual choice [42]. Our study adds to this literature and suggests that when caring for structurally vulnerable patients who use drugs, attributing patients’ unmet social needs as due to individual factors contribute to suboptimal social intervention. People who use drugs and are experiencing unstable and/or lack of housing often have personal histories and social circumstances which require social needs to be balanced with limited time and resources [43, 44]. The complexity of patients’ structural barriers can result in difficulties in providing compassionate care [45], which may in part explain the varied quality of social services provided to structurally vulnerable patients who use drugs. Participants in our study who were affiliated with the AMCT often held more structural views. This may be, in part, because the AMCT was established to provide care for patients who use drugs [34, 35]. AMCT staff may therefore be more familiar with structural barriers specific to this population compared to social service providers outside of the AMCT who provide care to a broader spectrum of patients and may spend less time working with patients who use drugs. Increasing recognition of systemic factors that shape substance use and unstable/lack of housing to broader groups of social service providers may help counter provider burnout and negative clinical interactions by increasing appreciation for patients’ circumstances [46, 47]. It may therefore be beneficial to provide formal structural competency training (i.e., training health professionals to recognize and respond to the impact of upstream, structural factors on patient health) [2, 48] for social service providers, especially for those with a more reductionist view (e.g., attributing patients’ unmet social needs due to individual factors such as motivation). While this type of training may increase understanding of structural factors and how to practically intervene on them, it is only a partial response to improving the overall care for this patient population. Structural competency training should be complemented with additional training on substance use and unstable/lack of housing, as well as rapport building and cultural safety [26].

While social service providers have identified constraints to addressing social needs within hospitals (e.g., limited resources, hierarchies, pressure to discharge) [45, 49, 50], our findings emphasize that the hospital environment is an opportunity to provide social services that are often difficult to access and maintain for structurally vulnerable patients. Hospitalization can temporarily alleviate some of the immediate structural vulnerabilities faced by patients (e.g., lack of shelter, food insecurity, acute withdrawal) [51, 52] and therefore provides a comparatively stable environment where social needs can be attended to without competing with other patient priorities. To take advantage of this brief window of opportunity, improvements need to be made to streamline social service provision. Neglecting to identify social needs limits the quality of care provided to patients [53], yet documentation of housing status [54, 55] and substance use [56] in acute care settings is inconsistent. Active case finding and tracking data on social determinants of health or using Bourgois et al.’s (2017) structural vulnerability assessment tool for clinical encounters may be an important first step in strengthening acute care’s role in social service provision. Screening for social needs and structural vulnerability should be complemented with broader culture change and care coordination. Doing so may ultimately increase quality of care, efficiency, prevent readmissions, improve successful discharges, and provide cost savings [57].

Complicating improvements to hospital care, however, are gaps in community-based supports for patients who use drugs and are experiencing unstable/lack of housing and have medical needs. Participants explained that the majority of community housing programs lack specialized medical care. This care gap is concerning because: 1) it can delay discharge or result in patients being turned away by housing supports, [12]; and 2) substance use is associated with higher odds of chronic and acute medical illnesses [58] which require tailored and often ongoing medical care. Our findings suggest that appropriate transitional housing programs, hospital-based Housing First teams, and substance use oriented sub-acute care facilities tailored for structurally vulnerable patients who use drugs and have other complex medical needs, could better meet the needs of patients experiencing hospital-community transitions. Providing patients experiencing unstable and/or lack of housing and medical illness with respite transitional housing and then rapidly moving them to permanent supportive housing has shown reductions in emergency department visits and hospital stays [59]. Moreover, a Housing First pilot project that provided integrated medical, psychiatric, and substance use care for people experiencing unstable/lack of housing, medical illness, and substance use found reductions in acute care and medical respite service utilization, and cost benefits [60]. While this pilot was not hospital-based per se*,* hospital-based Housing First teams may increase acute care efficiency as collaboration between Housing First teams and social service providers could occur on site. It is important to note, however, that successfully implementing in-hospital Housing First teams will require a simultaneous increase in availability of appropriate community housing supports.

Our study also outlines the potential utility of minimizing complex and restrictive eligibility criteria for income support policies. Previous research has also found that such policies function to compound existing structural vulnerabilities and ultimately create avoidable harms [61]. Increasing the amount of income support is also likely to be of benefit, especially since substance use creates additional subsistence needs beyond food and shelter (e.g., securing substances, medication, transportation costs). Importantly, our study highlights that while housing and income are necessary social needs, they are only one component of addressing structural vulnerability. Multi-level interventions that address intersecting factors are necessary to improve post-discharge outcomes and reduce admissions. For example, interventions that address other contextual factors (e.g., personal care skills, support systems) may help to mitigate structural factors that affect social service provision as well as patient outcomes once discharged and/or housed [62]. Increasing the availability of service models that couple provision of independent housing with on-site and community-based supports for intersecting issues (e.g. low-barrier, permanent supportive housing) may also be effective in improving long-term residential stability and health and social wellbeing [63, 64]. It is imperative that these initiatives ensure that substance-related health needs are addressed (e.g. through harm reduction, treatment and/or other support) along with housing and other structural factors.

### Limitations

To our knowledge, this study is the first to explicitly examine acute care social service providers’ perspectives on addressing the needs of structurally vulnerable patients who use drugs. This study included a novel mix of participants, incorporating the perspectives of social workers, peer support workers, and transition coordinators, ultimately broadening understanding of social service delivery within acute care hospitals. However, our study is not without limitations. Our focused ethnography targeted one large urban acute care hospital that operated a specialized team dedicated to caring for patients who use drugs and are experiencing unstable and/or lack of housing, which may not be representative of other acute care hospitals and constricts the relevance of our findings for other hospital settings. Our study was also time-limited and omitted participant observation. While this helped produce rapid data to generate practical recommendation, it limited the extent to which we could understand the full scope of social service provision from an observer standpoint. To protect participant confidentiality, we did not collect participant demographics and were unable to further break down ‘other social service providers’ into peer support workers and transition coordinators. Moreover, the small sample sizes between participant role types were not sufficient to conduct formal comparative analyses. As such, we were not able to provide further context on the participants themselves, which limits the transparency of the contrasting views presented in the theme ‘Conflicting views on patient-level barriers to care’. While we attempted to reduce potential investigator bias through several strategies (e.g., audit trail; reflexivity; team member review of coding, codebook, transcripts, and categorization; community advisory group consultation), latent content analysis requires coding the underlying context of participants’ accounts which requires subjective examination of the data. Moreover, coded transcripts were reviewed and not double coded by another team member. As such, it is still possible that investigator bias influenced our interpretation of the data, and in turn, our findings. Nevertheless, this study offers notable contributions. It produces new insights on how social services are provided to a patient population typically underserved in a setting not traditional to social services, and provides new insights to improve social service provision within acute care and post-discharge outcomes.

## Conclusions

Our findings revealed several barriers that limit the successful provision of social services within acute care for structurally vulnerable patients who use drugs, and suggest a number of acute care and broader policy changes that could potentially improve this population’s health and social wellbeing. While ambivalence over the role of the hospital and the reductionist views held by some social service providers themselves act as potential barriers to effective care, the hospital has the potential to serve a coordinated role in social service delivery. We suggest that acute care facilities augment their role as providers of social services and advocate for multi-level policy and interventions that address structural vulnerability, medical needs, and substance use.

## Supplementary Information

**Additional file 1.** Consolidated criteria for reporting qualitative studies (COREQ): 32-item checklist.

**Additional file 2.** Semi-structured interview guide.

## Data Availability

The dataset analysed for this study are not publicly available in order to protect participant anonymity. Additional information may be provided upon reasonable request to the corresponding author.

## Abbreviations

AMCT: Addiction medicine consult team
COREQ: Consolidated criteria for reporting qualitative studies
SSP: Social service provider
SW: Social worker
