# Supplementary material for: Social service providers’ perspectives on caring for structurally vulnerable hospital patients who use drugs: a qualitative study
Source: BMC Health Serv Res. 2022 Sep 8;22:1138. doi: 10.1186/s12913-022-08498-x (PMC9461250; doi:10.1186/s12913-022-08498-x)
Supplement: Supplementary file 1 — Additional file 1. Consolidated criteria for reporting qualitative studies (COREQ): 32-item checklist. [file 12913_2022_8498_MOESM1_ESM.docx]

**Additional file 1. Consolidated criteria for reporting qualitative studies (COREQ): 32-item checklist**

| **No. Item** | **Guide questions/description** | **Response** | **Reported on Page #** |
| --- | --- | --- | --- |
| **Domain 1: Research team and reﬂexivity** | | | |
| *Personal Characteristics* | | | |
| 1.Interviewer/facilitator | Which author/s conducted the interview or focus group? | EH and a research coordinator (AP) | 8 & Acknowledgements |
| 2. Credentials | What were the researcher’s credentials? E.g. PhD, MD | NG: MSc  KS: MSc  KD: MD, MSc  BP: PhD  GS: MD, MSc  EH: PhD | Title page |
| 3. Occupation | What was their occupation at the time of the study? | NG: MSc Student  KS: Research Coordinator  KD: Physician  BP: Professor  GS: Physician  EH: Assistant Professor | - |
| 4. Gender | Was the researcher male or female? | Female | - |
| 5. Experience and training | What experience or training did the researcher have? | NG: Trainee in qualitative research and previous qualitative experience  KS: Experience in qualitative research  KD: Experience in qualitative research  BP: Advanced experience in qualitative research  GS: Experience in qualitative research  EH: Advanced experience in qualitative research | - |
| *Relationship with participants* | | | |
| 6. Relationship established | Was a relationship established prior to study commencement? | AP has no prior relationship with participants. Given the close collaboration between our research group and the hospital, EH was previously acquainted with two participants. However, EH did not hold any influence over these participants or their employment status, and they were advised that their interview would be confidential. | 8 |
| 7. Participant knowledge of the interviewer | What did the participants know about the researcher? e.g. personal goals, reasons for doing the research | Participants were briefed on the purpose of the study. Ethics approval had been granted, participants reviewed the information sheet prior to written informed consent. | 8 |
| 8. Interviewer characteristics | What characteristics were reported about the interviewer/facilitator? e.g. Bias, assumptions, reasons and interests in the research topic | Author of this study have a particular interest in, and research, the topic of illegal drug use and harm reduction. | - |
| **Domain 2: study design** | | | |
| *Theoretical framework* | | | |
| 9. Methodological orientation and Theory | What methodological orientation was stated to underpin the study? e.g. grounded theory, discourse analysis, ethnography, phenomenology, content analysis | Focused ethnography, latent content analysis | 9-11 |
| *Participant selection* | | | |
| 10. Sampling | How were participants selected? e.g. purposive, convenience, consecutive, snowball | Personal invitations, flyer distribution, and presentations at hospital staff meetings. Interview participants also referred colleagues who might be interested in participating. | 8 |
| 11. Method of approach | How were participants approached? e.g. face-to-face, telephone, mail, email | Face-to-face, email | 8 |
| 12. Sample size | How many participants were in the study? | Of 28 potential participants who either referred to, or were contacted by, the study team, 10 were lost to follow-up and 18 provided informed consent and participated in a semi-structured interview. | 8 |
| 13. Non-participation | How many people refused to participate or dropped out? Reasons? | None | - |
| *Setting* | | | |
| 14. Setting of data collection | Where was the data collected? e.g. home, clinic, workplace | Private area of the hospital | 8 |
| 15. Presence of non-participants | Was anyone else present besides the participants and researchers? | No | - |
| 16. Description of sample | What are the important characteristics of the sample? e.g. demographic data, date | Participants were social workers (SW; n=8) and other social service providers (SSP; n=10), including peer support workers and transition coordinators. The ‘other’ category was used to protect participant anonymity for social service providers occupying otherwise identifiable positions | 9 |
| *Data collection* | | | |
| 17. Interview guide | Were questions, prompts, guides provided by the authors? Was it pilot tested? | Yes, semi-structured interview guide that was pilot tested. | 8 & Additional file 2 |
| 18. Repeat interviews | Were repeat inter views carried out? If yes, how many? | No | - |
| 19. Audio/visual recording | Did the research use audio or visual recording to collect the data? | Yes, audio-recorded. | 8 |
| 20. Field notes | Were ﬁeld notes made during and/or after the interview or focus group? | Yes, field notes and an audit trail | 8 |
| 21. Duration | What was the duration of the inter views or focus group? | Approximately 1 hour | 8 |
| 22. Data saturation | Was data saturation discussed? | Yes, no new ideas or concepts were emerging from interviews and preliminary analysis showed thematic saturation | 9 |
| 23. Transcripts returned | Were transcripts returned to participants for comment and/or correction? | Participants were given the option of reviewing and approving their transcript prior to inclusion in the analysis. | 8 |
| **Domain 3: analysis and ﬁndings** | | | |
| *Data analysis* | | | |
| 24. Number of data coders | How many data coders coded the data? | Two | 10 |
| 25. Description of the coding tree | Did authors provide a description of the coding tree? | Socioecological model | 10-11 |
| 26. Derivation of themes | Were themes identiﬁed in advance or derived from the data? | Derived from the data | 9-10 |
| 27. Software | What software, if applicable, was used to manage the data? | NVivo 12 | 9 |
| 28. Participant checking | Did participants provide feedback on the ﬁndings? | No, but the Inner City Health and Wellness Program’s advisory group of people with lived/living experience of substance use provided feedback on the findings. | 11 & Acknowledgments |
| *Reporting* | | | |
| 29. Quotations presented | Were participant quotations presented to illustrate the themes/ﬁndings? Was each quotation identiﬁed? e.g. participant number | Yes | 19-20 |
| 30. Data and ﬁndings consistent | Was there consistency between the data presented and the ﬁndings? | Yes | 19-20 |
| 31. Clarity of major themes | Were major themes clearly presented in the ﬁndings? | Yes | 19-20 |
| 32. Clarity of minor themes | Is there a description of diverse cases or discussion of minor themes? | Yes | 19-20 |

Developed from: Tong A, Sainsbury P, Craig J. Consolidated criteria for reporting qualitative research (COREQ): a 32-item checklist for interviews and focus groups. *International Journal for Quality in Health Care*. 2007. Volume 19, Number 6: pp. 349 – 357
