# Supplementary material for: Social service providers’ perspectives on caring for structurally vulnerable hospital patients who use drugs: a qualitative study
Source: BMC Health Serv Res. 2022 Sep 8;22:1138. doi: 10.1186/s12913-022-08498-x (PMC9461250; doi:10.1186/s12913-022-08498-x)
Supplement: Supplementary file 2 — Additional file 2. Semi-structured interview guide. [file 12913_2022_8498_MOESM2_ESM.docx]

**Additional file 2. Semi-structured interview guide**

*We are interviewing you today to find out more about what staff at the Royal Alexandra Hospital [RAH] think about homelessness and unstable housing amongst patients at the Royal Alexandra Hospital, and how the Addiction Recovery and Community Health [ARCH] team and the hospital could better address this issue. We are interviewing you because you were identified as someone who has been in direct or indirect contact with patients who are unstably housed or homeless, ARCH patients, and/or the ARCH team. We are interested in hearing your perspective on how social services are provided to unstably housed or homeless patients at the RAH, including the approaches ARCH and other social service providers (i.e. unit social workers, homeless transition coordinator) use to connect patients to housing, income, and other social supports. We would also like to hear your opinions on how ARCH and other social services providers could improve the social determinants of health for RAH patients who are homeless or unstably housed. This includes barriers and facilitators to improving care for ARCH patients and others who are unstably housed or homeless. Just a reminder before we start that no one outside of this room will be able to identify you based on what you say about the hospital or the ARCH team, so please be as open as possible.*

**Topic Area I: Experience with patients experiencing unstable housing or homelessness**

| **QUESTIONS:** | **PROBES:** |
| --- | --- |
| **Can you tell me how your position brings you into contact with patients who are unstably housed or homeless, ARCH patients, or the ARCH team?** | - What is your specific position and role? - How often do you encounter unstably housed or homeless patients? - How often do you encounter patients that are being seen by the ARCH Team? |
| **How does the hospital identify patients who are homeless or unstably housed?** | - What are barriers/facilitators to identifying unstably housed or homeless patients? - Are patients routinely asked about their housing? |
| **How can the RAH better track patients experiencing homelessness?** | - What would help units identify all patients in need of housing support? - How should housing status be captured in health records? |
| **What is your experience providing care to homeless or unstably housed patients?** | - Specific examples/incidents - How does providing care for an unstably housed, or homeless patients differ from providing care to other patients, if at all? - What makes it easy to care for patients who are homeless? What makes it difficult to care for patients who are homeless? |
| **What factors influence patient access to housing in the community?** | - Are there any obstacles that prevent patients from finding housing? - What kinds of resources are available in the community to help patients find housing? |

**Topic Area II: RAH model of social care**

| **QUESTIONS:** | **PROBES:** |
| --- | --- |
| **What kind of social work supports do RAH patients have access to?** | - How would you describe the RAH approach to supporting the social situation of patients? - Are these supports available to all RAH patients? - How does this differ for ARCH patients, if at all? - Probe for specific income, housing, identification, and other social supports? - How much emphasis is put on finding patients housing while they are hospitalized? |
| **Who provides social work supports to unstably housed or homeless RAH patients?** | - How often are Royal Alex social workers involved? - How often is the ARCH social worker involved? - How often is the Community and Social Services Homeless Transition Coordinator involved? - How often are other ARCH/hospital clinicians involved? - How often are staff from Housing First teams involved? - How often are social workers from community organizations involved? |
| **Are the social supports offered by the RAH the same or different than what would be available in the community? In what way?** | - How does hospital social work differ from social work practice in community settings? - Does the ARCH team offer any social work services that are not typically available? What are they? |
| **What is the impact of the social work provided at RAH on unstably housed or homeless patients?** | - Probes for: housing, health outcomes, social outcomes - How well does the current model meet patients’ social needs? - Are the impacts the same or different for ARCH patients, who also have access to an ARCH social worker? |
| **What is the impact of the RAH model of social care on you, as a [*position title*]? On other social service providers?** | - Does this model make your job more or less difficult? - Does this model make your colleagues work more or less difficult? |

**Topic Area III: Collaboration between RAH social work, community social work, and ARCH social work**

| **QUESTIONS:** | **PROBES:** |
| --- | --- |
| **How do RAH social workers collaborate with community-based social service providers?** | - Probe for their involvement/collaboration with, Homeless Transition Coordinator, Human Services staff, community-based social workers, Housing First team, etc. How would you describe the strengths of these collaborations? The weaknesses/challenges of these collaborations? - How does the hospital social worker/social support staff communicate and collaborate with a patient’s supports in the community? - How would you suggest these collaborations could be improved? |
| **How does the ARCH social worker collaborate with unit social workers?** | - How would you describe the strengths of these collaborations?  The weaknesses/challenges of these collaborations? - How would you suggest these collaborations could be improved? |
| **What value, if any, does the ARCH team social worker bring to RAH?** | - Strengths of this role? - Weaknesses of this role? - Are there other things the ARCH SW should be doing? - Can unit social workers fill the ARCH team social worker’s role? |
| **How would you change the role of the ARCH team social worker or the way they interact with other RAH staff? Patients?** | - What would you change about the ARCH’s model of patient care/model of social stabilization? - What would you change about the ARCH team social worker’s process? - What would you change about the way the ARCH team social worker communicates or collaborates with other RAH staff? |

**Topic Area IV: Improving RAH and ARCH social work services for patients who are unstably housed or homeless**

| **QUESTIONS:** | **PROBES:** |
| --- | --- |
| **Does it make sense to try and address homelessness in the acute care setting? Why or why not?** | - How might the RAH be uniquely placed to provide social work services that have been challenging to address in the community setting? |
| **How could the RAH hospital better meet the needs of patients who are unstably housed or homeless?** | - Probe for specific recommendations related to housing, income support, other social determinants of health |
| **What do you think is the biggest barrier to ending the practice of discharging patients to homelessness or unstable housing?** | - Other barriers? - What internal or external factors make it harder for the ARCH team or hospital social worker to succeed? - Are these threats/obstacles different or similar for ARCH patients vs. other unstably housed/homeless patients? |
| **What do you think is the biggest strength the RAH has in terms of   ending the practice of discharging patients to homelessness or unstable housing?** | - Other strengths? - What internal or external factors make it easier for hospital social workers to succeed? Are these the same for ARCH social workers? |
| **Do you see value in having a designated Housing First team that operates out of RAH and is for hospital patients? Why/why not?** | - What would this look like? - How would having a hospital-based Housing First team impact patient care? The broader community? |
| **What other strategies could help improve social work care at RAH for unstably housed or homeless patients?** | - Probe for specific initiatives related to housing, income support, other social determinants of health |
| **Is there anything else you would like to tell me about social work or unstable housing and homelessness at the RAH?** |  |
